# Pharmacological Behavior of Propylene Glycol/Polyvinyl Alcohol Hydrogel Incorporating Indomethacin Nanocrystals in the Skin

**DOI:** 10.3390/gels11040251

**Published:** 2025-03-27

**Authors:** Hiroko Otake, Fumihiko Ogata, Yosuke Nakazawa, Manju Misra, Masanobu Tsubaki, Naohito Kawasaki, Noriaki Nagai

**Affiliations:** 1 Faculty of Pharmacy, Kindai University, 3-4-1, kowakae, Higahi-Osaka 577-8502, Osaka, Japan; hotake@phar.kindai.ac.jp (H.O.); ogata@phar.kindai.ac.jp (F.O.); kawasaki@phar.kindai.ac.jp (N.K.); 2 Faculty of Pharmacy, Keio University, 1-5-30 Shibakoen, Tokyo 105-8512, Japan; nakazawa-ys@keio.jp; 3 Department of Pharmaceutics, National Institute of Pharmaceutical Education and Research, Opposite Air Force Station, Palaj Basan Road, Village Palaj, Gandhinagar 382355, Gujarat, India; manju.misra@lmcp.ac.in; 4 Graduate School of Pharmacy, Gujarat Technological University, Gandhinagar Campus Nr. Government Polytechnic K-6 Circle, E-4 Electronic Estate G.I.D.C, Sector-26, Gandhinagar 382028, Gujarat, India; 5 Laboratory of Pharmacotherapy, Kagawa School of Pharmaceutical Sciences, Tokushima Bunri University, 1314-1 Shido, Sanuki 769-2193, Kagawa, Japan; tsubaki@kph.bunri-u.ac.jp

**Keywords:** indomethacin, nanocrystal, hydrogel, propylene glycol, polyvinyl alcohol, transdermal drug delivery system

## Abstract

Background: We previously reported that carbopol hydrogels incorporating indomethacin nanoparticles (IMC NPs) improved the low permeability and bioavailability of skin formulations in transdermal drug delivery systems. However, the combination of NPs with other types of hydrogels has not been sufficiently explored to date. Therefore, this study investigated propylene glycol (PG)/polyvinyl alcohol (PVA) hydrogel as an alternative base to carbopol hydrogel for incorporating IMC NPs. Methods: IMC NPs were prepared using bead milling treatment, and these NPs were incorporated into PG/PVA hydrogel (IMC-NP@PG/PVA hydrogel). The IMC concentration was measured using the HPLC method, and seven-week-old Wistar rats were used to evaluate skin absorption. Results: Bead milling reduced the IMC particle size in the PG/PVA hydrogels to the nanoscale (30–200 nm) without altering its crystalline form. The IMC-NP@PG/PVA hydrogel exhibited enhanced uniformity, solubility, and drug release compared to the IMC microparticle-loaded PG/PVA hydrogel (IMC-MP@PG/PVA hydrogel), with a 1.44-fold greater area under the concentration–time curve. Transdermal permeability studies revealed that IMC-NP@PG/PVA had 2.36-fold higher absorption than the IMC-MP@PG/PVA hydrogel, with dissolved IMC permeating the skin. Pharmacokinetics in the rats showed significantly increased plasma levels, absorption rates, and bioavailability for IMC-NP@PG/PVA, demonstrating its superior delivery efficiency. Moreover, the skin absorption of IMC-NP@PG/PVA was higher than that of carbopol hydrogel. Conclusions: These findings highlight the potential of PG/PVA hydrogels as an effective base for transdermal drug delivery systems based on NPs.

## 1. Introduction

Indomethacin (IMC) is a widely used nonsteroidal anti-inflammatory drug (NSAID). IMC inhibits cyclooxygenase (COX) enzymes, particularly COX-2 enzymes, which play a key role in prostaglandin production, resulting in anti-inflammatory, analgesic, and antipyretic effects [1]. Thus, the administration of IMC is effective for managing pain and inflammation in various conditions such as joint disorders and arthritis; however, the oral administration of IMC is associated with significant limitations. Its weakly acidic nature and low water solubility contribute to a reduced bioavailability, while its short elimination half-life (approximately 4.5 h) necessitates frequent dosing, increasing the likelihood of gastrointestinal complications such as ulcers and bleeding [2]. These drawbacks have driven the development of alternative delivery strategies via skin pathways.

Systemic-action drugs (transdermal drug delivery system (DDS)), which penetrate the systemic circulation, provide advantages such as improved patient compliance, especially for individuals with gastrointestinal issues or those unable to tolerate oral medications. However, transdermal DDSs require the drug to infiltrate the skin tissue, and several barriers must be passed for this to be achieved. The skin, comprising the epidermis, dermis, and subcutaneous tissue, serves as a formidable barrier to drug permeation due to the stratum corneum (SC)—a layer rich in sebum, corneocytes, and intercellular lipids [3]. This barrier limits the transdermal absorption of most drugs, necessitating advanced formulation technologies to enhance permeability.

Numerous techniques have been developed to enhance transdermal drug absorption, such as absorption enhancers and iontophoresis [4,5,6]. These strategies offer significant advantages for the enhancement of absorption via skin pathways, although the SC continues to pose a substantial barrier to effective drug delivery. To overcome this limitation, recent innovations have focused on nanosizing drugs into nanoparticles (NPs) [7,8,9]. This approach has demonstrated several benefits, including enhanced transdermal permeability, strong adhesion to intercellular spaces due to electrostatic forces, improved membrane absorption, and faster dissolution rates [7,10]. These advancements afforded by nanosizing drugs are pivotal in optimizing transdermal DDSs for both localized and systemic therapeutic applications. Various types of NPs, including nanocrystals, lipid-based nanocarriers, and polymeric NPs, have demonstrated the ability to enhance drug stability and penetration [11]. Drug delivery via NPs utilizes multiple pathways, including intercellular lipid channels, transcellular mechanisms, and appendageal routes such as sweat glands and hair follicles, to improve bioavailability [7,10]. Importantly, NPs preserve the integrity of the skin barrier, thereby reducing the potential for irritation or damage often associated with conventional enhancement methods. It has been reported that these nanoscale carriers, typically ranging in size from 40 to 800 nm, interact with the lipid matrix of the SC, increasing the drug concentration in the deeper skin layers [7,8,9]. In our previous studies, we also successfully prepared dispersions containing IMC NPs by using wet bead milling with methylcellulose SM-4 as a stabilizer. Furthermore, our findings demonstrated that processing these dispersions containing IMC NPs significantly enhanced drug absorption through the skin compared to dispersions composed of microparticles (MPs) or dissolved formulations [11]. We also showed that particle size plays a role in the absorption of these drugs; improvements in drug absorption via the eye and intestine were observed with particles smaller than 400 nm, while drug absorption via the skin required particle sizes smaller than 200 nm, with the use of l-menthol as an additive [7]. These findings underscore the potential of NP-based transdermal delivery systems in optimizing the efficacy and safety of IMC and other pharmaceutical agents. In addition, we previously demonstrated that carbopol hydrogel exhibited excellent performance in facilitating the release of hydrophobic substances such as NPs from gel matrices [7]. However, to the best of our knowledge, the combination of NPs with other types of hydrogels has not been sufficiently explored to date.

Polyvinyl alcohol (PVA) is a hydrophilic polymer known for its biocompatibility and excellent film-forming properties. PVA hydrogels possess three-dimensional network structures formed by hydrophilic polymer chains, which create water-filled pores that facilitate the diffusion of solutes [12,13]. PVA is a semicrystalline, hydrophilic polymer characterized by extensive inter- and intra-molecular hydrogen bonding, which significantly affects its rheological and physical behavior [14]. While these hydrogen bonds provide excellent mechanical strength and film-forming capabilities, they also pose challenges during fabrication, including high viscosity and time-dependent solution properties. To enhance stability and address aging-related issues, PVA hydrogels are often stabilized through crosslinking, either chemically or physically [15,16,17,18,19]. Physical crosslinking is particularly beneficial for medical applications due to its elimination of toxic intermediates. Propylene glycol (PG), classified as Generally Regarded as Safe (GRAS) by the FDA, serves as a plasticizer, improving the flexibility and mechanical properties of hydrogels, and it is a widely used biocompatible plasticizer for PVA [14,20,21]. The addition of PG effectively modifies PVA’s properties by disrupting hydrogen bonds, which reduces viscosity and improves mechanical characteristics such as toughness, elongation at break, and ductility, thereby expanding PVA’s versatility across various applications [14,21]. Thus, the PG/PVA-based hydrogel, like carbopol, is a water-soluble gel. On the other hand, it is expected that the higher viscosity of PG/PVA hydrogel compared to carbopol likely enhances interfacial adhesion with the skin and formulations, promoting deeper penetration of NPs and consequently improving transdermal absorption.

In this study, we examined whether PG/PVA-based hydrogels incorporating IMC NPs are useful as base materials for transdermal DDSs.

## 2. Results and Discussion

### 2.1. Changes in Drug Particle Size in PG/PVA Hydrogel Incorporating IMC with or Without Bead Milling

**Figure 1:**
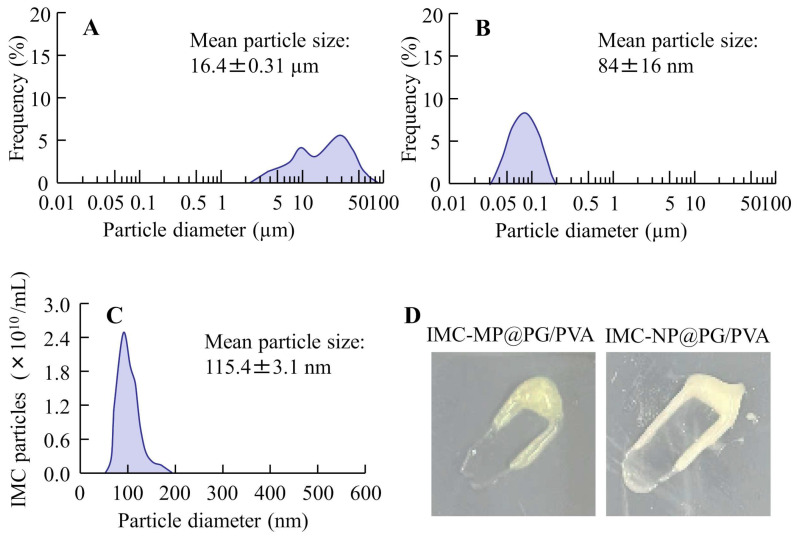
Particle size frequency and images of IMC-MP@PG/PVA and IMC-NP@PG/PVA hydrogels. The particle size distribution data of the IMC-MP@PG/PVA hydrogel (**A**) and IMC-NP@PG/PVA hydrogel (**B**) determined with SALD-7100. (**C**) The particle size distribution of the IMC-NP@PG/PVA hydrogel determined using NANOSIGHT LM10. (**D**) Digital photos of the IMC-MP@PG/PVA and IMC-NP@PG/PVA hydrogels. Particle size was significantly reduced via bead milling, and the mean particle size of the IMC-NP@PG/PVA hydrogel was 30–200 nm.

We previously demonstrated that carbopol hydrogels incorporating IMC NPs exhibit high skin permeability and systemic absorption [11]. In this study, we prepared IMC NPs incorporated into PG/PVA hydrogel as an alternative to carbopol hydrogel, and we investigated how this modification of the hydrogel base affects the release, skin permeability, and systemic absorption of IMC NPs. PVA is a hydrophilic polymer known for its biocompatibility and excellent film-forming properties, and PG serves as a plasticizer, improving the flexibility and mechanical properties of hydrogels [12,13,14,21]. It is known that hydrogels with PG can modulate drug release rates [12,13,14,21], and PVA and PG have been fabricated/formulated as a hydrogel with applications in various fields such as biomedical devices, DDSs, wound dressings, and tissue engineering. Based on this background, we selected PG/PVA hydrogel as the gel base material for NPs. Additionally, the choice of additives during the preparation of NPs is critically important. It is well known that, without the addition of methylcellulose SM-4 during wet milling, poorly soluble drugs and water molecules entangle, forming a meringue-like structure [22]. Therefore, the inclusion of methylcellulose SM-4 during the wet milling process is essential. Furthermore, the addition of 2-hydroxypropyl-β-cyclodextrin (HPβCD) enhances the stability of NPs in dispersions [22]. Moreover, it is known that NPs with particle sizes exceeding 100 nm cannot penetrate the skin in the absence of l-menthol; l-menthol allows for the skin penetration of 200 nm particles [7,11]. Thus, the addition of l-menthol is also crucial for developing skin-applicable formulations. Taken together, we attempted to prepare a PG/PVA hydrogel incorporating IMC NPs by using bead milling and various additives (methylcellulose SM-4, HPβCD, and l-menthol). Figure 1 shows images and the particle sizes of the PG/PVA hydrogel incorporating IMC NPs. The IMC MP-loaded PG/PVA hydrogel (IMC-MP@PG/PVA hydrogel) exhibited visible yellow particles of IMC dispersed within the gel matrix. In contrast, the IMC NP-loaded PG/PVA hydrogel (IMC-NP@PG/PVA hydrogel) looked like a white, uniformly scattered system, with IMC particles showing a homogeneous distribution. The particle size of the IMC powder was 16.4 ± 0.34 μm, and bead milling decreased the IMC to nanosize. The particle sizes of the IMC-NP@PG/PVA hydrogels determined using SALD-7100 and NANOSIGHT LM10 were 84 ± 16 nm and 115.4 ± 3.1 nm, respectively.

**Figure 2:**
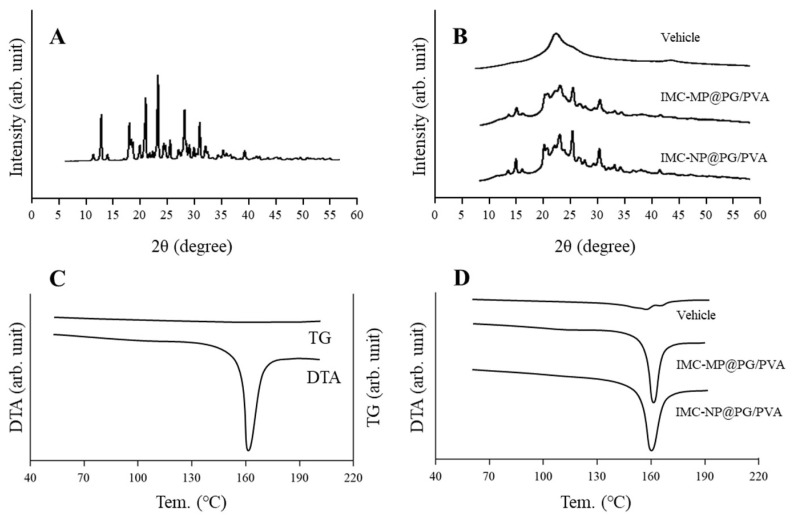
XRD and TG-DTA patterns of the IMC-MP@PG/PVA and IMC-NP@PG/PVA hydrogels. (**A**) XRD pattern of the IMC powder. (**B**) Changes in XRD pattern of the vehicle, IMC-MP@PG/PVA hydrogel, and IMC-NP@PG/PVA hydrogel. (**C**) TG and DTA patterns of the IMC powder. (**D**) Changes in DTA pattern of the vehicle, IMC-MP@PG/PVA hydrogel, and IMC-NP@PG/PVA hydrogel. The melting point of the IMC powder, IMC-MP@PG/PVA hydrogel, and IMC-NP@PG/PVA hydrogel was 162.99 °C, 162.56 °C, and 162.77 °C, respectively. The peak of the melting point was not detected in the vehicle. No difference in the XRD or DTA patterns were observed between the IMC-MP@PG/PVA and IMC-NP@PG/PVA hydrogels.

Additionally, we examined the crystalline structure by using powder X-ray diffraction (XRD) and a thermogravimetry–differential thermal analysis (TG-DTA). The XRD and TG-DTA patterns of the IMC particles in the IMC-MP@PG/PVA and IMC-NP@PG/PVA hydrogels were similar (Figure 2). These data suggest that the crystalline structure remained unchanged post-milling, and they show that bead milling effectively reduced IMC to the nanoscale, achieving a homogeneous dispersion within the PG/PVA hydrogels while preserving its crystalline structure.

### 2.2. Evaluation of Physical Properties of PG/PVA Hydrogel Incorporating IMC NPs

**Figure 3:**
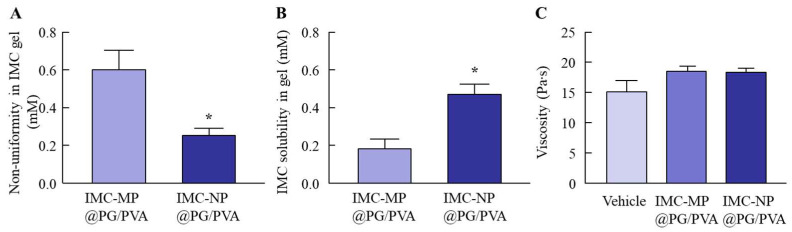
Non-uniformity (**A**), solubility (**B**), and viscosity (**C**) of the IMC-MP@PG/PVA and IMC-NP@PG/PVA hydrogels. n = 5. * *p* < 0.05 vs. IMC-MP@PG/PVA hydrogel for each category (Student’s *t*-test). The uniformity and solubility of the IMC in the IMC-NP@PG/PVA hydrogel were higher than those of the IMC in the IMC-MP@PG/PVA hydrogel. However, the viscosity of the IMC hydrogel was similar in the IMC-MP@PG/PVA and IMC-MP@PG/PVA hydrogels.

**Figure 4:**
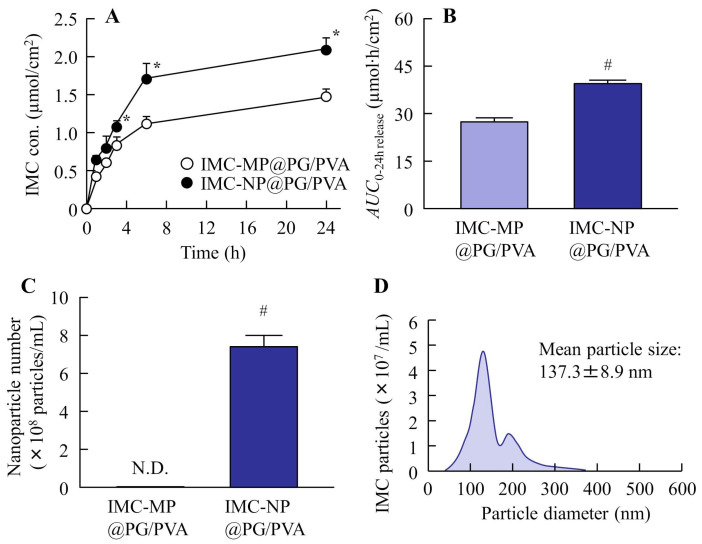
IMC release behavior from the IMC-MP@PG/PVA and IMC-NP@PG/PVA hydrogels through a 450 nm pore membrane. Drug release (**A**) and *AUC*_0–24h release_ (**B**) from the IMC-MP@PG/PVA and IMC-NP@PG/PVA hydrogels. Number (**C**) and size distribution (**D**) of the IMC NPs in the reservoir chamber 24 h after the application of the IMC-NP@PG/PVA hydrogel. n = 5. N.D., not detectable. * *p* < 0.05 vs. IMC-MP@PG/PVA hydrogel for each category (Tukey–Kramer test). ^#^ *p* < 0.05 vs. IMC-MP@PG/PVA hydrogel for each category (Student’s *t*-test). The drug release from the IMC-NP@PG/PVA hydrogel was higher than that from the IMC-MP@PG/PVA hydrogel, and IMC NPs (40–360 nm) were detected in the reservoir chamber after the application of the IMC-NP@PG/PVA hydrogel.

Figure 3 show the changes in the physical properties of the PG/PVA hydrogel incorporating IMC with or without bead milling. The uniformity and solubility of the IMC-NP@PG/PVA hydrogel were significantly higher than those of the IMC-MP@PG/PVA hydrogel. However, the IMC-MP@PG/PVA and IMC-NP@PG/PVA hydrogels had similar viscosity, with a value of approximately 18.5 Pa∙s. Figure 4 shows the drug release profiles of the IMC-MP@PG/PVA and IMC-NP@PG/PVA hydrogels in the in vitro study using a Franz diffusion cell with a 0.45 μm membrane filter. The release of IMC from the IMC-NP@PG/PVA hydrogel was higher than that from the IMC-MP@PG/PVA hydrogel, with the area under the concentration–time curve (*AUC*_0–24h release_) being 1.44-fold higher than that of the IMC-MP@PG/PVA hydrogel. Furthermore, the IMC particles released from the IMC-NP@PG/PVA hydrogel were measured to be in the range of 40–360 nm, confirming the release of NPs. In contrast, particles smaller than 1 µm were not detected in the IMC-MP@PG/PVA hydrogel. These results show that some IMC was released from the IMC-NP@PG/PVA hydrogel as NPs.

### 2.3. Skin Absorption of IMC in PG/PVA Hydrogel Incorporating IMC NPs

**Figure 5:**
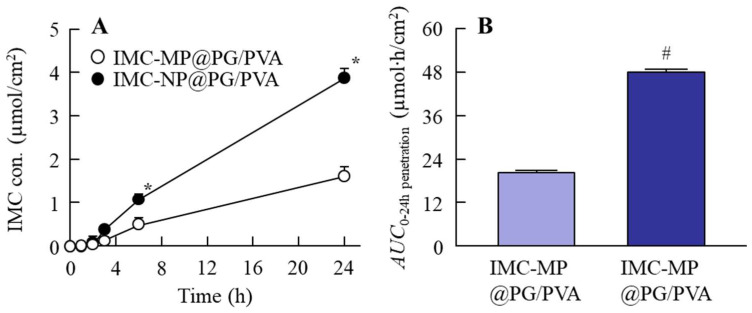
Changes in transdermal penetration of IMC from the IMC-MP@PG/PVA and IMC-NP@PG/PVA hydrogels. Transdermal penetration (**A**) and *AUC*_0–24 penetration_ (**B**) of the IMC-MP@PG/PVA and IMC-NP@PG/PVA hydrogels. n = 5. * *p* < 0.05 vs. IMC-MP@PG/PVA hydrogel for each category (Tukey–Kramer test). ^#^ *p* < 0.05 vs. IMC-MP@PG/PVA hydrogel for each category (Student’s *t*-test). The drug penetration of the IMC-NP@PG/PVA hydrogel was higher than that of the IMC-MP@PG/PVA hydrogel. In contrast to the results obtained for the release of IMC from the IMC-NP@PG/PVA hydrogel, IMC NPs were not detected in the reservoir chamber after application.

Figure 5 shows the transdermal permeability profiles of the IMC-MP@PG/PVA and IMC-NP@PG/PVA hydrogels in the in vitro study using a Franz diffusion cell with rat skin, and Table 1 shows the kinetic parameters summarizing the data in Figure 5, obtained using Equations (1) and (2). The penetration of IMC from the IMC-NP@PG/PVA hydrogel was higher than that from the IMC-MP@PG/PVA hydrogel, with *AUC*_0–24h penetration_ being 2.36-fold higher than that of the IMC-MP@PG/PVA hydrogel. The *J*_c_, *K*_p_, and *K*_m_ of the IMC-NP@PG/PVA hydrogel also significantly increased in comparison with the IMC-MP@PG/PVA hydrogel. However, no significant differences were observed in *τ* and *D* between the two formulations. In contrast to the results of the experiments using membranes, all IMC particles that permeated through the skin were in a dissolved state in the experiments using rat skin, and no nanosized IMC particles were detected on the reservoir side. It was suggested that the IMC permeating the rat skin was entirely dissolved, as no nanosized particles were detected in the reservoir chamber in the transdermal permeability study.

**Figure 6:**
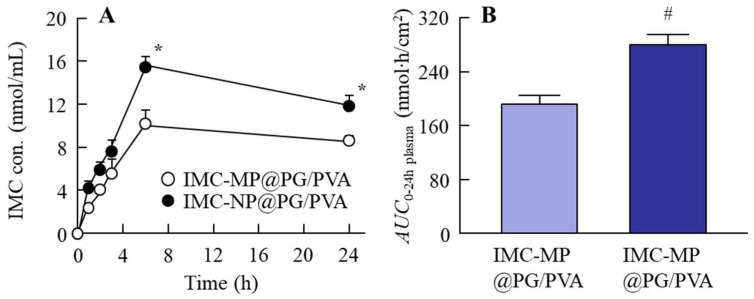
Changes in IMC levels in the blood of rats treated with the IMC-MP@PG/PVA and IMC-NP@PG/PVA hydrogels. Percutaneous absorption (**A**) and *AUC*_0–24 plasma_ (**B**) from the IMC-MP@PG/PVA and IMC-NP@PG/PVA hydrogels. n = 5. * *p* < 0.05 vs. IMC-MP@PG/PVA hydrogel for each category (Tukey–Kramer test). ^#^ *p* < 0.05 vs. IMC-MP@PG/PVA hydrogel for each category (Student’s *t*-test). The plasma IMC levels of the rats treated with the IMC-NP@PG/PVA hydrogel were higher than those of the rats treated with the IMC-MP@PG/PVA hydrogel.

Figure 6 shows the changes in the plasma concentration of rats treated with the IMC-MP@PG/PVA and IMC-NP@PG/PVA hydrogels, and Table 2 shows the kinetic parameters summarizing the data in Figure 6, obtained using Equations (3) and (4). The plasma IMC levels of the rats treated with the IMC-NP@PG/PVA hydrogel were also significantly higher than those of the rats treated with the IMC-MP@PG/PVA hydrogel, with *AUC*_0–24h plasma_ being 1.46-fold higher than for the IMC-MP@PG/PVA hydrogel. In addition, *k*_a_ and *F* were enhanced in the IMC-NP@PG/PVA hydrogel in comparison with the IMC-MP@PG/PVA hydrogel. Based on these results, it was hypothesized that the IMC NPs released from the hydrogel penetrated the skin and dissolved in the dermal layers. Subsequently, they were transported throughout the body via the bloodstream. Thus, the IMC-NP@PG/PVA hydrogel may improve transdermal permeability and systemic absorption compared to the IMC-MP@PG/PVA hydrogel, indicating its potential for enhanced drug delivery.

### 2.4. Comparison of Skin Absorption of PG/PVA Hydrogel and Previously Reported Carbopol Hydrogel Incorporating IMC NPs

**Figure 7:**
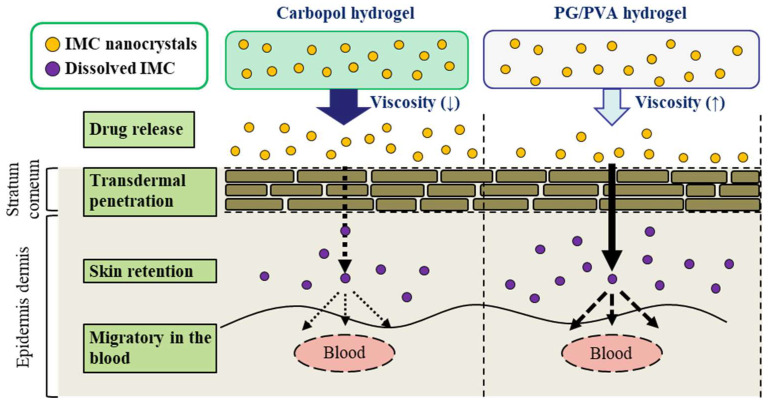
Scheme of skin absorption after treatment with the IMC-NP-loaded carbopol hydrogel and the IMC-NP@PG/PVA hydrogel. The application of the PG/PVA hydrogel supported the absorption of the IMC NPs and enhanced skin penetration and plasma IMC levels in comparison with the application of the carbopol hydrogel.

It is critically important to compare the in vivo pharmacokinetics of carbopol and PG/PVA hydrogels serving as the base for transdermal formulations incorporating drug NPs. Although NPs were released from both the PG/PVA and carbopol hydrogels, the drug release from the PG/PVA hydrogels (*AUC*_0–24h release_ 39.5 µmol·h/cm^2^, Figure 4A) was lower than that from the carbopol hydrogels in previous studies [11] (*AUC*_0–24h release_ 62.0 µmol·h/cm^2^). In contrast, the viscosity of the PG/PVA hydrogels was found to be 3.68-fold higher than that of the carbopol hydrogels (5.0 Pa∙s), suggesting that the higher viscosity may have influenced the drug release from the base [11]. However, the *K*_m_ of the IMC-NP@PG/PVA hydrogel was significantly higher than that of the carbopol hydrogel incorporating IMC NPs, and both the transdermal permeability and the plasma concentration in the rats treated with the IMC-NP@PG/PVA hydrogel were also significantly enhanced in comparison with the rats treated with the corresponding carbopol hydrogel (Table 1). These results may be led by the stronger bonding interface with the skin of the PG/PVA hydrogel in comparison with carbopol hydrogels and support the findings that the drug was released from the PG/PVA hydrogels, enabling effective NP delivery (Figure 7). Additional research is required to clarify the transdermal pathways of drug NPs within PG/PVA. NPs are internalized through multiple mechanisms, including passive diffusion, receptor-mediated endocytosis, and phagocytosis/micropinocytosis. Previous studies have consistently shown that the size of NPs significantly affects their endocytosis [23,24,25]. Consequently, future investigations will explore the connection between drug absorption and endocytic processes.

## 3. Conclusions

**Figure 8:**
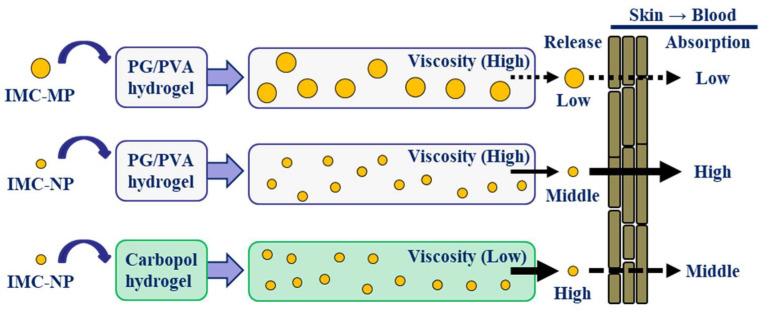
Scheme of skin absorption process in IMC-loaded hydrogels discussed this study.

We prepared an IMC NP-loaded PG/PVA hydrogel. The PG/PVA hydrogel effectively incorporating IMC NPs demonstrated promising properties as a base for transdermal drug delivery. Compared to previously reported carbopol hydrogels [11], the PG/PVA hydrogels exhibited lower drug release but higher viscosity. Additionally, the PG/PVA hydrogels incorporating IMC NPs achieved significantly enhanced transdermal permeability, skin partition, and systemic absorption of IMC NPs, with superior pharmacokinetic performance in comparison with IMC-MP@PG/PVA hydrogels and corresponding carbopol gels (Figure 8). These findings highlight the potential of PG/PVA hydrogels for effective NP delivery, providing a viable platform for improving transdermal drug formulations.

## 4. Materials and Methods

### 4.1. Reagents

All reagents used were of analytical grade or the highest available purity. Briefly, ten percent ammonia solution (molecular weight (MW) 17.03, purity 28.0–30.0%), PVA (MW 1500–1800, saponification degree 78–82 mol%), PG (MW 1500–1800, purity > 99.0%), isoflurane (MW 184.49, purity > 98.0%), propyl p-hydroxybenzoate (MW 180.20, purity > 95.0%), heparin (MW 100,000 units, activity 130 units/mg), l-menthol (MW 156.27, purity > 98.0%), and IMC powder (MW 357.79, purity > 98.0%) were sourced from Wako Pure Chemical Industries Ltd. (Osaka, Japan). Methylcellulose SM-4 (mean MW 15,000, purity > 98.0%) was procured from Shin-Etsu Chemical Co. Ltd. (Tokyo, Japan). HPβCD (MW 1500, purity 98.0%) was provided by Nihon Shokuhin Kako Co., Ltd. (Tokyo, Japan). Additionally, pentobarbital (MW 248.29, purity > 98.0%) from Tokyo Chemical Industry Co., Ltd. (Tokyo, Japan), and a Bio-Rad Protein Assay Kit (Bio-Rad, Hercules, CA, USA) were used.

### 4.2. Animals

Twenty-five male rats, 7 weeks of age and weighing around 200 g, were obtained from Kiwa Laboratory Animals Co., Ltd. (Wakayama, Japan). The animals were categorized into five groups (5 × 5 = 25) to evaluate in vitro transdermal penetration (5 × 2 = 10) and skin absorption (5 × 3 = 15) using PG/PVA hydrogels incorporating IMC MPs and IMC NPs. The rats were maintained under standard environmental conditions (light cycle: 7:00–19:00; dark cycle: 19:00–7:00; ambient temperature: 25 °C) and fed with free access to CE-2 (CLEA Japan, Inc., Tokyo, Japan, commercial feed) and water. All animal procedures complied with the guidelines of Kindai University, the Japanese Pharmacological Society, the National Institutes of Health Guide for the Care and Use of Laboratory Animals, and the ARRIVE (Animal Research: Reporting of In Vivo Experiments) guidelines. Moreover, euthanasia was carried out by administering pentobarbital (200 mg/kg, i.p.) in accordance with the 2020 AVMA guidelines [26]. The experimental protocol received approval from the Kindai University Animal Experimental Committee on 1 April 2022 (approval number: KAPS-2022-017).

### 4.3. PG/PVA Hydrogels Incorporating IMC MPs and IMC NPs

Hydrogels incorporating IMC MPs and NPs were prepared using a modified breakdown method previously reported [7,11]. First, 0.15 g of IMC powder (commercial product) was blended with methylcellulose SM-4, and the total volume was adjusted to a 10 mL HPβCD solution to create IMC MP dispersions. Additionally, another dispersion was prepared by grinding 0.15 g of IMC powder in an agate mortar and pestle for 60 min, mixing it with methylcellulose SM-4, and diluting it to a 10 mL HPβCD solution. For the preparation of the IMC NP dispersions, the dispersions were transferred to a 2 mL tube (TOMY Seiko Co., Ltd., Tokyo, Japan) incorporating 2 g of zirconia beads (0.1 mm in diameter), filling the tube to about 80% of its capacity. The mixture was then subjected to milling using a Shake Master Neo (1500 rpm, Biomedical Science Co., Ltd., Tokyo, Japan) for 3 h.

In this study, we prepared 1% IMC hydrogels incorporating IMC MPs and IMC NPs (IMC-MP@PG/PVA and IMC-NP@PG/PVA hydrogels) using the procedures described below. The 1% IMC-loaded PG/PVA hydrogels (IMC-MP@PG/PVA and IMC-NP@PG/PVA hydrogels) were prepared as follows: First, 264 μL of PG were added to 26 μL of purified water and heated to 90 °C using a mini-block bath (MyBL-10). Subsequently, 180 μL of PVA were added, and the mixture was heated for 30 min. After heating, 1 mL of the IMC dispersion was incorporated. Additionally, to prepare the IMC-MP@PG/PVA and IMC-NP@PG/PVA hydrogels, 0.03 g of the transdermal absorption enhancer l-menthol was added. For l-menthol preparation, 0.05 g of l-menthol was dissolved in 500 μL of ethanol in a Petri dish and recrystallized by allowing the ethanol to evaporate at room temperature. Table 3 shows the compositions of the IMC-MP@PG/PVA and IMC-NP@PG/PVA hydrogels. These gels were stable for at least 7 d at room temperature.

### 4.4. Measurement of IMC Levels Using HPLC

The measurement of IMC was conducted using an HPLC system, specifically the LC-20AT (Shimadzu Corp., Kyoto, Japan) [11]. Chromatographic separation was achieved with a mobile phase composed of acetonitrile and 0.05 M acetic acid in a 70:30 (*v*/*v*) ratio, flowing at a rate of 0.25 mL/min. For the hydrogel samples, dilution with methanol was performed, and, subsequently, centrifugation was conducted at 20,400× *g* for 20 min at 4 °C. Blood samples were processed under the same centrifugation conditions. The supernatants obtained from these procedures were analyzed to quantify the IMC levels in the blood. In each analysis, 100 µL of the sample were pipetted into a vial, and 50 µL of 3 µg/mL methanolic propyl p-hydroxybenzoate in methanol (serving as the internal standard) were added. A 10 µL portion of this prepared mixture was injected using an SIL-20AC auto-sampler. Chromatographic separation was performed at 35 °C on an Inertsil^®^ ODS-3 column maintained in a CTO-20AC oven (GL Science Co., Inc., Tokyo, Japan), with detection carried out at a wavelength of 254 nm. The IMC peak appeared at 10.1 min, while the internal standard peak emerged at 3.7 min. The limit of detection (LOD) and limit of quantitation (LOQ) for IMC were determined to be 39.6 ng/mL and 120 ng/mL, and the intra- and inter-day accuracy were 99.3% and 98.6%, respectively (n = 6). The intra- and inter-day of relative standard deviation (RSD) were 0.61% and 1.09%, respectively (n = 6). The accuracy and RSD were calculated by using 5 µg/mL IMC and an internal standard.

### 4.5. Characterization of IMC Particles in PG/PVA Hydrogels

The particle size, distribution, and crystalline state of the IMC in the PG/PVA hydrogels were evaluated using multiple techniques. For the particle size and distribution analyses, the hydrogels were diluted 1000-fold with purified water and assessed using two instruments: a laser diffraction particle size analyzer, SALD-7100 (Shimadzu Corp., Kyoto, Japan), and a nanoparticle tracking system, NANOSIGHT LM10 (Quantum Design Japan, Tokyo, Japan). The SALD-7100 measurements were conducted with the light-scattering intensity set to 40–60% of the maximum and a refractive index of 1.60 ± 0.10i. For the NANOSIGHT LM10 analysis, particles were tracked under a blue laser (405 nm) for 60 s, with a solution viscosity set to 1.27 mPa·s [7,11]. The crystalline structure of the IMC in the hydrogels was determined through lyophilization and a subsequent analysis. The IMC hydrogels were diluted ten-fold with purified water and freeze-dried using a FREEZE DRYER FD-1000 (TOKYO RIKAKIKAI Co., Ltd., Tokyo, Japan) under conditions of −20 °C, 20 Pa pressure, and a 48 h drying period. The lyophilized samples were examined using XRD and TG-DTA. The XRD analysis was performed on a Mini Flex II diffractometer (Rigaku Co., Tokyo, Japan), with diffraction angles scanned between 5° and 60° at a rate of 10°/min. TG-DTA was carried out using a DTG-60H simultaneous thermal analyzer (Shimadzu Corp., Kyoto, Japan) with approximately 5 mg of sample under a nitrogen atmosphere, with the temperature increasing from 50 °C to 200 °C at a rate of 10 °C/min.

### 4.6. Evaluation of IMC@PG/PVA Hydrogel Properties

The IMC hydrogels (IMC-MP@PG/PVA and IMC-NP@PG/PVA hydrogels) were characterized by evaluating viscosity, drug solubility, and content uniformity [27]. Viscosity measurements were conducted using an SV-1A viscometer (A&D Company, Limited, Tokyo, Japan). Approximately 0.3 g of each hydrogel was analyzed at a rotational speed of 60 rpm and a temperature of 22 °C for 3.5 min. To determine the solubility of the IMC in the hydrogels, each formulation was diluted ten-fold with purified water at 22 °C. The soluble and insoluble IMC fractions were separated via centrifugation at 100,000× *g* using a Beckman Optima™ MAX-XP Ultracentrifuge (Beckman Coulter, Osaka, Japan). The IMC concentrations in the supernatants were quantified through HPLC [27]. The uniformity of the IMC distribution in the hydrogels was assessed by dividing 0.3 g of each formulation into 10 equal portions (0.03 g each). Each portion was dissolved in methanol and filtered through a 450 nm membrane, and the IMC content in the filtrates was quantified using HPLC. The standard deviation of the IMC content across the 10 portions was calculated to determine the distribution consistency within the hydrogel matrix.

### 4.7. In Vitro Drug Release and Transdermal Penetration Studies of IMC@PG/PVA Hydrogel

In vitro assessments of IMC release and transdermal penetration were performed using Franz diffusion cells (effective diffusion area: 2 cm^2^) under controlled conditions. For drug release studies, a DURAPORE membrane filter (0.45 µm pore size) was affixed to the diffusion cell. The receptor chamber (capacity: 12.2 mL) was filled with 0.2 mM phosphate buffer (pH 7.2) and maintained at 37 °C with continuous magnetic stirring to ensure uniformity. A 0.3 g aliquot of either the IMC-MP@PG/PVA or the IMC-MP@PG/PVA hydrogel was applied to the donor compartment, which was sealed with a stopper and wrapped in aluminum foil to prevent evaporation and contamination. At predetermined intervals (1, 2, 3, 6, and 24 h), 200 µL aliquots were withdrawn from the receptor chamber and replaced with fresh buffer. Each sample (50 µL) was combined with 100 µL methanol, and the IMC concentration was determined via HPLC. Particle size distribution was assessed using a NANOSIGHT LM10 system. The *AUC*_0–24h release_ was calculated using the trapezoidal rule up to 24 h [7].

For transdermal penetration studies, abdominal skin comprising SC, epidermis, and dermis from ten seven-week-old Wistar rats (allocated into two groups of five) was prepared by shaving 24 h before the experiment. The rats were euthanized with pentobarbital (200 mg/kg), and the excised skin was mounted onto a Franz diffusion cell. The receptor chamber (12.2 mL) was filled with 0.2 mM phosphate buffer (pH 7.2) and maintained at 37 °C. A 0.3 g portion of the IMC hydrogel was uniformly spread on the skin surface. At designated intervals (1, 2, 3, 6, and 24 h), 100 µL aliquots were sampled from the receptor chamber for HPLC and NANOSIGHT LM10 analyses. The transdermal penetration area under the concentration–time curve (*AUC*_0–24h penetration_) was calculated using the trapezoidal rule. Additionally, pharmacokinetic parameters, including the penetration rate (*J*_c_), skin/preparation partition coefficient (*K*_m_), penetration coefficient (*K*_p_), diffusion constant (*D*), lag time (*τ*), skin thickness (*δ*, 0.071 cm), and cumulative drug amount (*Q*_t_), were determined according to established equations (Equations (1) and (2)) [7]:

(1) $$J_{c}=\frac{Q}{A\cdot \left(t-\tau \right)}=\frac{D\cdot K_{m}\cdot C_{c}}{\delta }{=K}_{p}\cdot C_{c}$$

(2) $$D=\frac{\delta^{2}}{6\tau }$$

### 4.8. Percutaneous Absorption Studies of IMC@PG/PVA Hydrogel

To investigate IMC systemic absorption, experiments were conducted using seven-week-old male Wistar rats. Fifteen rats were separated into three groups (five rats each). Jugular vein cannulation was performed under isoflurane anesthesia (flow rate: 1.0 L/min; concentration: 3%). A 1.5 cm incision was made on the neck, and the external jugular vein was exposed. A catheter pre-filled with heparin solution (10 IU/mL) was inserted approximately 3 cm into the vein, secured with ligatures, and externalized through the dorsal neck. After a 24 h recovery period, 0.3 g of IMC hydrogel was uniformly applied to a 2 cm^2^ area of the abdominal skin comprising SC, epidermis, and dermis. Blood samples (200 µL) were collected via the cannula at specified intervals (1, 2, 3, 6, and 24 h). The collected blood samples were centrifuged at 24,000 × *g* for 20 min at 4 °C, and the resulting plasma was analyzed to determine the IMC concentration via HPLC. These operations were performed as described in a previous study [11]. The area under plasma IMC concentration–time curve (*AUC*_0–24h plasma_) was computed using the trapezoidal rule up to 24 h. Subsequently, pharmacokinetic parameters were derived using Equations (3) and (4), as described in previous studies [7]:

(3) $$C_{\mathrm{IMC}}{=C}_{0}\cdot e^{{-k}_{e}\cdot t}$$

(4) $$C_{\mathrm{IMC}}=\frac{k_{a}\cdot F\cdot D}{V_{d}\left(k_{a}{-k}_{e}\right)}\left(e^{{-k}_{e}\cdot \left(t-\tau \right)}-e^{{-k}_{a}\cdot \left(t-\tau \right)}\right)$$

The elimination rate constant (*k*_e_) and volume of distribution (*V*_d_) were determined through Equation (3), following the intravenous administration of 0.3 mL of IMC solution (200 µg/kg) into the femoral vein. The initial plasma concentration (*C*_0_), *k*_e_, and *V*_d_ were calculated as 2.68 ± 0.13 µg/mL, 0.05 ± 0.07 h^−1^, and 52.1 ± 1.98 mL/kg, respectively (n = 5). For the percutaneous absorption analysis, the absorption rate constant (*k*_a_) and bioavailability (*F*) were estimated using Equation (4).

### 4.9. Statistical Analysis

All statistical analyses were performed using JMP software version 5.1 (SAS Institute, Cary, NC, USA). The results are expressed as mean values with the corresponding standard error of the mean. Group comparisons were assessed via the unpaired Student’s *t*-test and one-way repeated-measures analysis of variance (ANOVA). When significant differences were detected by the ANOVA, post hoc analyses were conducted using the Tukey–Kramer test. A *p*-value of less than 0.05 was regarded as indicative of statistical significance.

## Figures and Tables

**Table 1:** Pharmacokinetic analysis of IMC@PG/PVA in in vitro transdermal penetration.

Formulation	*J*_c_ (µmol/cm^2^/h)	*K*_p_ (×10^−3^ cm/h)	*K*_m_ (×10^−1^)	*τ* (h)	*D* (×10^−3^ cm^2^/h)
IMC-MP@PG/PVA	0.38 ± 0.04	1.37 ± 0.02	1.86 ± 0.17	1.61 ± 0.13	0.52 ± 0.02
IMC-NP@PG/PVA	0.91 ± 0.04 *	3.30 ± 0.19 *	4.54 ± 0.39 *	1.64 ± 0.13	0.51 ± 0.04

n = 5. * *p* < 0.05 vs. IMC-MP@PG/PVA for each category (Student’s *t*-test).

**Table 2:** Pharmacokinetic analysis of percutaneous absorption of IMC@PG/PVA.

Formulation	IMC-MP@PG/PVA	IMC-NP@PG/PVA
*k_a_* (h^−1^)	0.31 ± 0.05	0.39 ± 0.05
Bioavailability *F* (×10^−1^)	0.12 ± 0.01	0.26 ± 0.01 *

The *k*_e_ was 0.05 ± 0.07 h^−1^. N = 5. * *p* < 0.05 vs. IMC-MP@PG/PVA for each category (Student’s *t*-test).

**Table 3:** Compositions of IMC@PG/PVA in this study.

Formulation	IMC	Methylcellulose	HPβCD	PG	PVA	l-Menthol	Purified Water ad.	Treatment
IMC-MP@PG/PVA	1 g	0.5 g	5 g	17.6 mL	12 g	2 g	100 g	—
IMC-NP@PG/PVA	1 g	0.5 g	5 g	17.6 mL	12 g	2 g	100 g	Bead mill

## Data Availability

The data generated in this study can be requested from the corresponding author.

## Abbreviations

The following abbreviations are used in this manuscript:

ANOVA	one-way repeated-measures analysis of variance
COX	cyclooxygenase
DDS	drug delivery system
DTA	differential thermal analysis
HPβCD	2-hydroxypropyl-β-cyclodextrin
IMC	indomethacin
MP	microparticle
NP	nanoparticle
NSAID	nonsteroidal anti-inflammatory drug
PG	propylene glycol
PVA	polyvinyl alcohol
RSD	relative standard deviation
SC	stratum corneum
TG	thermogravimetry
XRD	powder X-ray diffraction
